# How job crafting behaviors influence the innovative behavior of knowledge workers in the gig economy: based on the organismic integration theory

**DOI:** 10.3389/fpsyg.2023.1228881

**Published:** 2023-09-04

**Authors:** Linpei Song, Sung Jun Jo

**Affiliations:** School of Business Administration, Gachon University, Seongnam, Republic of Korea

**Keywords:** job crafting strengths, job crafting interests, controlled motivation, autonomous motivation, innovative behavior

## Abstract

**Introduction:**

The gig economy is extolled for its potential to stimulate economic and social development. This study examines the mediating roles of controlled and autonomous motivation in the relationship between job crafting and innovative behavior in the context of knowledge workers in the gig economy.

**Methods:**

To examine these relationships, we propose and test a conceptual framework using an online survey conducted among knowledge workers in China. The participants consisted of 302 knowledge workers who voluntarily participated in the study. We used structural equation modeling to test the proposed relationships among the variables.

**Results:**

Controlled and autonomous motivation mediates the relationship between job crafting and innovative behavior.

**Discussion:**

Our study shed light on the knowledge workers’ motivation dilemma in the gig economy, with theoretical implications for research regarding job crafting, motivation, and practice implications about the job crafting and innovative behavior of knowledge workers.

## 1. Introduction

“Gig job” primarily refers to unconventional job types carried out by gig workers who exhibit adaptability, diversity, and innovation. In China, over 400 million people are expected to join the gig economy within the next 15 years, according to a projection by the Ali Research Institute (Zheng and Yang, 2020). Gig jobs are available, and then they allow knowledge workers to practice their innovative ideas (Shah et al., 2022; Xuan, 2022; Knight et al., 2022), which is essential for organizations to remain competitive, differentiate themselves from rivals, and achieve a competitive advantage in today’s fast-paced and constantly shifting business environment (Alfy and Naithani, 2021). The majority of research on the gig economy has focused on low-skilled occupations, such as consumer-led services, transportation, and delivery services (Duggan et al., 2020). However, additional research on highly skilled knowledge workers in the gig economy is required. Gig economy enables high-skilled knowledge-based gig workers to establish new relationships with businesses based on their strengths and interests (Wilkins et al., 2022). Innovative behavior is the contribution of individuals and groups to the development of new services, products, tasks, or ideas for the achievement of desired results (Watson et al., 2021). This is especially crucial in the gig economy, where workers must frequently be self-motivated and adaptable to succeed. “Gig job” makes extensive use of the Internet and mobile technology to quickly match supply and demand, as opposed to the traditional scheduled work (Friedman, 2014). As non-traditional forms of employment increase, the benefit of job crafting that enables knowledge workers to increase their sense of autonomy and innovativeness has emerged. However, knowledge workers’ motivation and innovation behavior in the gig economy have become crucial for enterprises, and more research is required in this area.

Since gig jobs allow employees to practice innovative ideas with additional income, they can contribute to job crafting behavior, which can lead to increased job motivation.

We trace the highlights of proactive perspectives that reflect the increasing significance of employees taking initiative to anticipate and initiate changes in the manner in which work is performed in response to rising levels of uncertainty and dynamism. Knowledge workers can create employment across industries and professions by utilizing their strengths and interests, which is also beneficial for boosting motivation. Nevertheless, the effect of job crafting on motivation and innovative behavior was frequently reported as weaker than anticipated (Duggan et al., 2020; Wilkins et al., 2022). This is due to the fact that the majority of previous research has focused on top-down interventions in job design, which have only addressed the superficial representation of job crafting (Zhang et al., 2021). It does not consider how to incorporate employees’ motivations, strengths, and interests into the job crafting concept in order to cultivate motivation and creativity (Berg et al., 2013). Modifying employment characteristics based on the initiative of the individual is the premise of job crafting (Parker et al., 2001). Top-down interventions emphasized task standardization and simplification to increase work motivation and innovation. This strategy was criticized for its counterproductive nature and disregard for employee motivation (Taneja et al., 2011; Parker et al., 2017). The concept of job crafting was devised to address these issues on the basis of motivation theories (Deci and Ryan, 2008; Deci and Ryan, 2012; Herzberg, 2017; Ryan et al., 2021). In addition to research on the various forms of job crafting, research on innovative behavior requires studies on motivational processes. The motivational process enables knowledge workers to adapt and operate effectively in the face of an ever-changing stream of opportunities and threats within the gig economy.

Prior research on organizations has broadly linked innovative behavior of gig workers to either intrinsic or extrinsic motivation (Wong, 2019). In the organization literature, however, the effects of controlled and autonomous motivation on the formulation of innovative behavior have been neglected. Controlled motivation includes both external regulation (performing activities for rewards or to avoid punishments) and introjected regulation (performing activities in response to internal pressures such as shame, guilt, or pride) (Kooij et al., 2017; Zhang et al., 2021). In contrast, autonomous motivation (identification, integration, and intrinsic) refers to behaviors that are performed out of personal endorsement, volition, or preference (Kooij et al., 2017; Zhang et al., 2021). Knowledge workers’ controlled motivation in the gig economy may be the result of job crafting under pressure from the economy. While knowledge workers become autonomously motivated when they feel that the job’s objectives are consistent with their own values. The two aforementioned motivations may be present when knowledge-based workers craft their jobs in the gig economy.

Using organismic integration theory (OIT) concepts, this study aims to expand our understanding of the causes and effects of employee motivation in order to overcome the limitations of previous research. The OIT is suitable for this study because it focuses on internalizing and integrating extrinsic motivation. Utilizing OIT as a lens, this study proposes modifications to how knowledge-based employees craft their jobs by using their strengths and interests to increase motivation and account for innovative behavior.

For this objective, the following research questions will be investigated:

What is the relationship between job crafting and motivation among gig economy knowledge workers?

What is the relationship between motivation and innovative behavior in the gig economy among knowledge workers?

Does motivation serve as a mediator between job crafting and innovative behavior among gig economy knowledge workers?

## 2. Theoretical background

### 2.1. Organismic integration theory

As the core of biological, cognitive, and social regulation, motivation has been an essential topic of study in the field of psychology (Ryan and Deci, 2000). Motivation is also of particular importance with regard to individual differences in development rate and extent (Deci et al., 1985). Self-determination theory (SDT) is a metatheory of human motivation. The objective is to examine the interaction between extrinsic forces or factors acting on people (e.g., grades, evaluations, or payment) and the intrinsic motivations or needs inherent to humans (e.g., interests, curiosity, or enjoyment) (Deci et al., 1985; Ryan and Deci, 2000). OIT, a secondary theory of SDT proposed by Deci et al. (1985), Ryan and Deci (2020), emphasizes the process of internalizing external motivation more than SDT. After decades of development, OIT has been implemented in numerous fields, including subordinates’ knowledge creation behavior (Ma et al., 2023), students’ motivation (Wang et al., 2017), and immoral behavior (Orth et al., 2022). However, there needs to be more research on using OIT to explain how different forms of job crafting affect the innovative behaviors of knowledge workers in the gig economy.

According to OIT, the types of motivation in the middle of the continuum were reclassified as follows: external regulation and introjection represent controlled motivation, while identification, integration, and intrinsic motivation represent autonomous motivation (Deci et al., 2017). External regulation undermines autonomy and intrinsic motivation; introjected regulation is low in autonomy, while identification is more autonomous; Integration is the most autonomous form of extrinsic motivation, and when fully internalized and integrated, it does not necessarily become intrinsic motivation (Ryan and Deci, 2014). According to OIT, people are naturally active, and internal structures formed as a result of experience help shape behavior to some extent (Toward an Organismic Integration Theory, 2023). In the gig economy, for instance, the job content of knowledge workers is initially purely extrinsic; they may not be intrinsically motivated (not enjoy doing the job). However, while working on a task, they may value the job more and more, and it may become part of themselves, thereby being gradually internalized. With controlled motivation, the reasons for engaging knowledge workers in job crafting are unrelated to the job itself; it is rather instrumental in achieving enhanced personal economic benefits. On the other hand, identified and intrinsic motivation (autonomous motivation) entails understanding the motivations behind a person’s behavior, so they are autonomous (Van Den Broeck et al., 2016). And these types of motivational regulation are arranged along a continuous scale known as the self-determination continuum.

In the meantime, employees are required to promote unremarkable behaviors; they must consider not only how to prompt the behaviors, but also how to promote self-regulation of the behaviors so that they persist over time. The dynamic nature of behavioral regulations should be considered when facilitating the adoption of exercise behavior (Wasserkampf and Kleinert, 2016). Recent theories have, to varying degrees (Sheldon and Prentice, 2019; Ryan et al., 2021), continued to embrace such suggestions, recognizing the inherent tendencies of people to engage in active, curiosity-based exploration and to integrate new experiences into the self.

OIT can help explain the relationship between a person’s degree of controlled and autonomous motivation and their innovative behavior. The four external motivation subtypes of OIT (extrinsic, introjected, identified, and integrated regulation) in conjunction with intrinsic motivation in the domain of knowledge workers can be used to explain how individual job construction leads to innovative behavior. SDT, particularly OIT, has almost never been applied to the domain of knowledge workers before, which is one of the literature gaps identified by this study. According to (Locke and Schattke, 2019), the majority of research on extrinsic motivation in SDT has focused on the negative effects of eradicating incentives. In addition, they criticize the idea that extrinsic motivators are frequently portrayed as controlling and advocate for a framework that is more differentiated. In the literature on knowledge workers and in informal discussions among innovative behavior researchers, SDT has been consistently and generically referred to without this distinction between nuanced sub-theories. Consequently, the organismic integration process has rarely been investigated as an explanation of the mechanism by which job fabrication leads to innovative behavior via controlled and autonomous motivation. This original contribution serves as the foundation for the second research query.

### 2.2. Job crafting

Wrzesniewski et al. (2013) found that not much research has been done on job crafting behaviors that specifically try to change job tasks so that they fit the employee’s personal resources. Kooij et al. (2017) defined job crafting as the initiative of workers to tailor their work to their strengths (crafting to better align one’s personal strengths) and interests (crafting to engage in activities of personal interests). Wrzesniewski et al. (2013) both suggested that job-crafting behaviors should focus on employees’ motivations, strengths, and interests to create a better person-job fit. The fundamental tenet of job crafting is that people modify specific elements of their jobs or job roles on their own initiative in order to better match their careers with their skills, strengths and interests (Lepanjuuri et al., 2018). According to Wong et al. (2021), interests crafting may be easier for employees, as it requires less self-awareness than strengths of job crafting. Therefore, job crafting interests will make it easier for knowledge workers to match work resources in the gig economy, increase their motivation, and facilitate their innovative behavior. However, little empirical research has been conducted in this area, so its full scope is unknown (Lepanjuuri et al., 2018). Modern workplaces require knowledge workers’ (Engineers, Scientists, Information Technologists, Accountants, Researchers, Social Workers, etc.) specialized knowledge or skills; therefore, it’s essential to fill the gap of knowledge workers in the gig economy (Wong, 2019). To keep pace with these important and rapid changes, work design theory and research are undergoing a transformation.

### 2.3. Job crafting and motivation

Job design perspectives focus on how workers interpret task characteristics and social information to form work-related motivations. If job crafting provides workers with autonomy and the chance to express themselves, they may be more motivated to resist organizational constraints (Wrzesniewski and Dutton, 2001). By crafting their jobs, employees are able to better connect with the ultimate fruits of their labor and their beneficiaries, thereby making their work more meaningful and motivating (Berg et al., 2013). Previous researchers argued that jobs should not be simplified but rather crafted to motivate people to produce improved results (Gallagher and Einhorn, 1976; Pane Haden et al., 2012). Modern society requires a more comprehensive perspective that identifies new forms of job crafting in order to comprehend how various types of job crafting relate to motivation (Bruning and Campion, 2018). In this study, the motivation of knowledge workers is defined within OIT along a continuum of self-determination-related motivational quality. Through this process of integration, endowed with an inherent desire to pursue and develop their interests, people tend to seek out challenges, discover new perspectives, and actively internalize and transform practices (Ryan and Deci, 2002). When acting out of autonomous motivation, the reasons for knowledge workers engaging in job crafting are intrinsic enjoyment and interest in accomplishing tasks (Bureau et al., 2022). The highest degree of self-determination is represented by this. According to Bureau et al. (2022), given that achieving self-determination requires both high levels of autonomous motivation and low levels of controlled motivation, the fact that the theory’s main antecedents do not correspond to a central type of motivation raises the question of what circumstances cause knowledge workers to become more or less externally regulated (controlled motivation). In the context of the gig economy, there is reason to believe that knowledge workers will have work motivation after job crafting, whether their interests/strengths drive their autonomous motivation or their interests/strengths drive their controlled motivation. Therefore, it is crucial to consider the strengths and interests of knowledge workers when crafting employment and fostering motivation and innovative behavior.

### 2.4. Job crafting and autonomous motivation

The characteristic of autonomous motivation is that individuals engage in activities with free will and motivation (Deci et al., 2017). Typically, self-directed activities are intrinsically motivated. When individuals comprehend the value and purpose of their work, experience a sense of ownership and autonomy in its execution, and receive clear feedback and support, they are more likely to behave appropriately. A global shift from manufacturing economies to service and knowledge economies has profoundly altered the nature of work in organizations (Grant and Parker, 2009). These changes include a transition from a manufacturing-based to a service-based economy, an increase in the scope and significance of the knowledge-based industry, and “knowledge workers” who are subjected to rigorous cognitive demands (Grant and Parker, 2009). According to empirical studies conducted by Rockmann and Ballinger (2017), workers who provide professional services over the telephone have relatively high levels of control and autonomy over their work. Increasing numbers of knowledge workers are entering the labor force with specialized skills, interests, and prerequisites. In the years since work design theories entered the limelight, the nature of work has changed dramatically. Further study on knowledge workers’ work designs in the gig economy is required. According to eminent scholars, the work design theories that resulted from these efforts are part of a select group of organizational approaches that are simultaneously valid, significant, and useful (Miner, 1984; Miner, 2003). Few studies have been conducted to classify the various types of job crafting in the context of the knowledge economy (Kosenkranius et al., 2020; Irfan et al., 2022; Khan et al., 2022; Zhao et al., 2022). Strengths or interests that might encourage job crafting in the context of knowledge-based work in the gig economy are the subject of this study. We chose job crafting strengths and job crafting interests that would be particularly important for autonomous motivation in knowledge-intensive work: knowledge workers frequently implement job crafting based on their knowledge since they are highly respected for their specialized knowledge or skill. And the job crafting definition is expressed in terms of job demands to address the operationalization challenges of the role-based definition (Khan et al., 2022). This study begins with job crafting strengths, which are defined as the extent to which the job requires expert knowledge to solve complex problems. Increased specialized knowledge or skill might necessitate excellent expert understanding at work and foster autonomy by requiring greater independent problem-solving. Job crafting interests refers to the process of increasing knowledge workers’ senses of autonomous motivation by identifying interesting aspects of a job in order to promote innovative behavior.

In short, it can be said that knowledge employees are more likely to undertake job crafting based on their strengths or interests for autonomous motivation. In light of the above given theoretical and empirical support, the following hypotheses are developed.

H1: Job crafting strengths have a positive effect on autonomous motivation.

H2: Job crafting interests have a positive effect on autonomous motivation.

### 2.5. Job crafting and controlled motivation

Since COVID-19, many jobs have become more complex and uncertain, necessitating that employees continuously craft their employment and innovate to adapt to an ever-changing workplace (Ingusci et al., 2021). COVID-19 has had a negative impact on numerous real economies, which has resulted in the loss of a sizable number of jobs (Irfan et al., 2022). Due to economic pressure, many employees are compelled to (controlled motivation) customize their employment. These striking changes in the work environment require the development of new theoretical perspectives to aid scholars and practitioners in describing, explaining, and altering the nature of work (Barley and Kunda, 2001; Parker et al., 2001; Rousseau and Fried, 2001; Johns, 2006). Researchers now acknowledge that jobs vary not only in terms of core task characteristics, but also in terms of knowledge characteristics such as job complexity, information processing, problem-solving, and specialization among knowledge workers (Parker and Wall, 1998; Morgeson and Campion, 2003; Morgeson and Humphrey, 2006). Due to the increased uncertainty of work, proactive perspectives are more important when motivational approaches to job design from organizational psychology are considered. As opposed to the historically narrower focus on employment, there is now a greater emphasis on how knowledge-based work is organized. According to Chua and Iyengar (2006), Schwartz (2000), autonomy and demands increase, knowledge workers may experience tension and depression as a result of an excessive number of options and tasks to prioritize. This could negatively affect motivation and performance. On the other hand, according to research conducted by Gillet et al. (2013), controlled motivation correlates positively with job satisfaction. In other words, individuals construct their work based on their unique characteristics (strengths or interests) to maintain their physical and mental health despite the stress of their jobs (Bruning and Campion, 2018). Advances in work design theory and research are crucial for attaining the ideal balance. Motivational approaches to job design from organizational psychology, such as the OIT theory, a sub theory of the SDT theory, expand the definition of motivation to include both autonomous and controlled motivation. OIT maintains that job demand enhances internal motivation and internalization, resulting in greater accomplishments. Nevertheless, regardless of the type of job crafting, it occupies a specific position in the process of internalizing external motivation. Therefore, it is possible to hypothesize that job crafting can increase controlled motivation. Important distinctions must therefore be made between autonomous motivation and controlled motivation, as well as their relative difference or absence, as individuals with different absolute levels of autonomous motivation and controlled motivation may obtain the same difference or relative relationship (Brunet et al., 2015). Consequently, the following hypotheses have been formulated:

H3: Job crafting strengths have a positive effect on controlled motivation.

H4: Job crafting interests have a positive effect on controlled motivation.

### 2.6. Motivation and innovative behavior

According to the OIT, which has been applied and refined in multiple contexts over the past few years (Deci et al., 2017), individuals experience a spectrum of motivations, ranging from controlled to autonomous. According to OIT, there is a distinction between the relative autonomy of different motivation forms, such as autonomous motivation and controlled motivation, which promote performance on complex or creative tasks and behaviors (Bureau et al., 2022). Wong (2019) discovered a positive correlation between autonomous and controlled motivation in the context of knowledge employees. This demonstrates that knowledge worker work motivation is not always characterized by high levels of autonomous motivation and low levels of controlled motivation (Bureau et al., 2022). According to the findings of a meta-analysis by Van Den Broeck et al. (2021), controlled motivation (external regulation and introjection) and autonomous motivation (identification, integration, and intrinsic motivation) were positively associated with work performance. It has become essential to investigate the antecedents of these types of motivational experiences in order to promote work behaviors (innovative behavior) in organizations (Guo, 2018; Hope et al., 2019; Tang et al., 2021; Autin et al., 2022). Innovative behavior necessitates trial and error and the acceptance of failure as a stepping stone to learning; autonomous motivation enables knowledge workers to repeatedly test out new concepts. In the meantime, knowledge workers will activate controlled motivations to engage in innovative behaviors in order to maintain their employment. Previous research suggested that setting clear job tasks would be intrinsically motivating for workers and increase their innovation (Miao et al., 2022). Motivation has traditionally been regarded as a crucial factor in influencing employee innovation behavior, and it is frequently triggered by work tasks. In contrast to Jabagi et al. (2019), we propose that autonomous and controlled motivation will encourage innovative behavior among knowledge workers. In order to become more motivated, workers can switch up their work tasks. Employees who actively craft their jobs may have motivation for innovation (Wrzesniewski and Dutton, 2001). Since innovative behavior contributes to organizational innovation in the form of novel processes, it is worthwhile to examine its motivational antecedents (Saether, 2019). Consequently, the following hypotheses have been formulated:

H5: Autonomous motivation has a positive effect on innovative behavior.

H6: Controlled motivation has a positive effect on innovative behavior.

H7: Autonomous motivation has a mediating effect on the relationship between Job crafting strengths and innovative behavior.

H8: Autonomous motivation has a mediating effect on the relationship between job crafting interests and innovative behavior.

H9: Controlled motivation has a mediating effect on the relationship between Job crafting strengths and innovative behavior.

H10: Controlled motivation has a mediating effect on the relationship between job crafting interests and innovative behavior.

**Figure 1:**
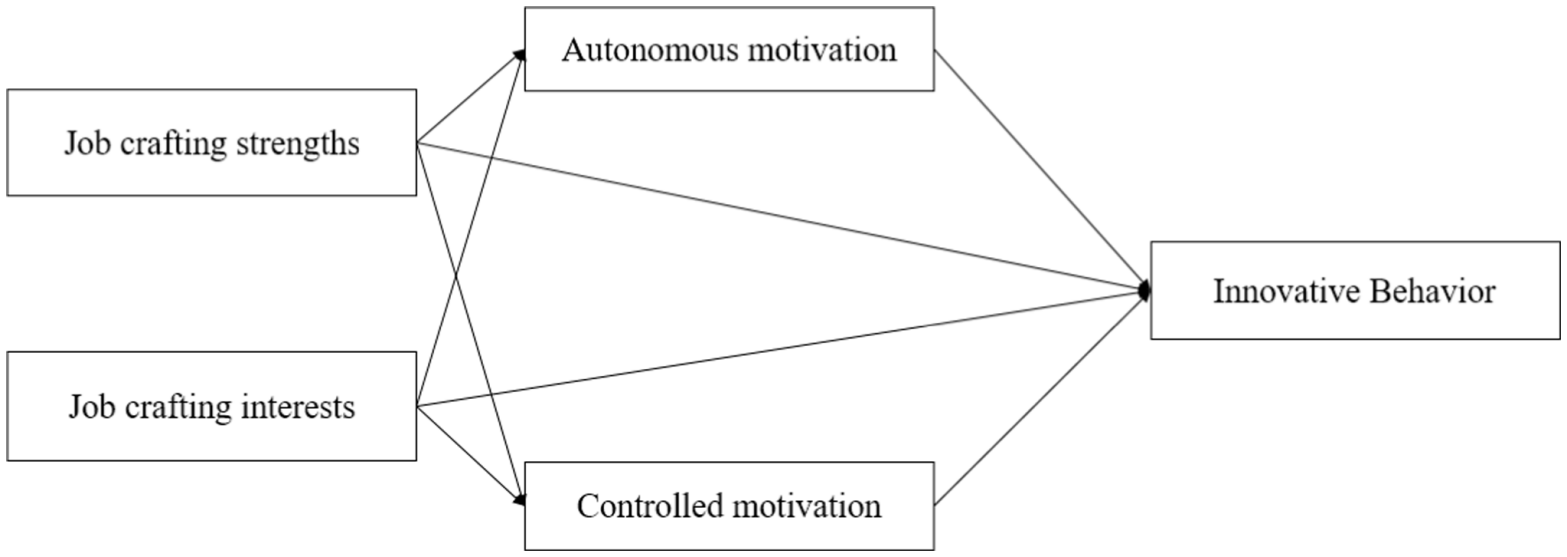
Conceptual model.

This study developed a theoretical framework based on the above hypotheses (Figure 1).

## 3. Method

### 3.1. Data collection

This study conducted an online questionnaire using the WENJUANXING survey platform (one of the most popular survey platforms in China) between 1 and 31 January 2023. Knowledge workers (such as engineers, teachers, designers, etc.) in China were the target population. Through the human resource (HR) departments of 20 Chinese firms, we identified and distributed the questionnaire link to qualified knowledge workers. Due to the inaccessibility of all employee databases, a sampling of convenience techniques was employed to collect data from knowledge workers. First, we defined knowledge workers and stated the purpose of this study. We reassured respondents of their anonymity during the survey and explained how our research could increase motivation for knowledge worker’s job crafting and innovative behavior in the gig economy.

The *a priori* sample size calculator for structural equation models was used to determine the required sample size. Using Free statistics calculators 4.0, anticipated effect size = 0.30 (medium), power level = 0.90, number of latent variables = 9 (this study has secondary dimensions), number of observed variables = 27, and probability = 0.05 were applied, and it was shown that a recommended sample size of 226 was required in the analysis of the current study (Structural equation model). However, the current study included a greater number of subjects to increase the statistical power as well as cope with the possibility of non-response error.

A set of sample from 302 knowledge workers were obtained, including unreliable responses. Excluding unreliable responses, a total of 283 responses were collected and used for analysis, with a response rate of 93.71%. The demographics of the respondents are presented in Table 1. More than half the respondents were male (*N* = 187, 66.1%), the majority of the respondents were between the ages of 31 and 35 (*N* = 132, 46.6%). Regarding education level, most respondents held a master’s degree (*N* = 143, 50.5%). The majority of the respondents were educators. The highest monthly income of 8,001–9,500 yuan was 27.9%, followed by 6,501–8,000 yuan, and 11,000 yuan above in third place.

**Table 1:** Demographic Information.

Demographic	Characteristic	Frequency	Percentage
Gender	Male	187	66.1%
	Female	96	33.9%
Age	25–30 years old	66	23.3%
	31–35 years old	132	46.6%
	36–40 years old	54	19.1%
	40 years old above	31	11.0%
Education	Junior college degree	11	3.9%
	Bachelor degree	75	26.5%
	Master degree	143	50.5%
	Doctoral degree	54	19.1%
Occupation	Instructor	153	54.1%
	Computer programmer	76	26.9%
	Designer	17	6.0%
	lawyer	21	7.4%
	Others	16	5.7%
Monthly income	5,000 yuan below	11	3.9%
	5,001–6,500 yuan	32	11.3%
	6,501–8,000 yuan	65	23.0%
	8,001–9,500 yuan	79	27.9%
	9,501–11,000 yuan	32	11.3%
	11,000 yuan above	64	22.6%

### 3.2. Measurement

The questionnaire included five constructs: job crafting strengths, job crafting interests, autonomous motivation, controlled motivation, and innovative behavior. Following the back-translation procedure suggested by Guo (2018), we used the original scales in English and then translated all of the items into Chinese. Specifically, items were translated into Chinese by a bi-lingual translator before being translated back into English by a second translator to ensure high clarity and accuracy. All measures were rated using a 5-point Likert scale (1: strongly disagree-5: strongly agree).

#### 3.2.1. Job crafting strengths

Job crafting strengths was a 4-item scale adapted from Zhang et al. (2021). The items include “I organized my work in such a way that it matches my strengths,” “In my work tasks I tried to take advantage of my strengths as much as possible,” and “I looked for possibilities to do my tasks in such a way that it matches my strengths.” The Cronbach’s *α* is 0.87.

#### 3.2.2. Job crafting interests

Job crafting interests was a 5-item scale adapted from Zhang et al. (2021). The items include “I actively looked for tasks that match my own interests,” “I organized my work in such a way that I could do what I find interesting,” and “I made sure that I take on tasks that I like.” The Cronbach’s *α* is 0.83.

#### 3.2.3. Autonomous motivation

Autonomous motivation (identification and intrinsic motivation) was measured by a 6-item scale adapted from Gagné et al. (2010). The identification items include “I chose this job because it allows me to reach my life goals,” “Because this job fulfills my career plans,” and “Because this job fits my personal values.” The intrinsic motivation items include “Because I enjoy this work very much,” “Because I have fun doing my job,” and “For the moments of pleasure that this job brings me.” The Cronbach’s *α* of identification is 0.83, intrinsic is 0.89.

#### 3.2.4. Controlled motivation

Controlled motivation (external regulation and introjection) was measured by a 6-item scale adapted from Gagné et al. (2010). The external regulation items include “Because this job affords me a certain standard of living,” “Because it allows me to make a lot of money,” and “I do this job for the paycheck.” The introjection items include “Because I have to be the best in my job, I have to be a ‘winner’,” “Because my work is my life and I do not want to fail,” and “Because my reputation depends on it.” The Cronbach’s *α* of external regulation is 0.69, introjection is 0.75.

#### 3.2.5. Innovative behavior

Innovative behavior was a 6-item scale adapted from Scott and Bruce (1994). Sample items are “While working, I have come up with innovative and creative notions,” “While working, I try to propose my creative ideas and convince others,” and “While working, I seek new service techniques, methods, or techniques.” The Cronbach’s *α* is 0.89.

### 3.3. Analytical procedures

In this study, descriptive analysis presented the demographic. Cronbach’s alpha was utilized to assess the reliability of each variable using SPSS 25.0. Confirmatory factor analysis (CFA) was used to examine the convergent and discriminant validity of the five main variables. The values of *χ*^2^/df, the comparative fit index (CFI), the Tucker-Lewis index (TLI), the root mean square error of approximation (RMSEA), and the root mean square residual (RMR) were examined to assess the overall model fit. The relationship between every variable was presented using correlation analysis. Harman one factor analysis was used to test the common method variance. We used structural equation modeling (SEM) to test the hypotheses in AMOS 24.0. Bootstrapping was set to 5,000 resamples for mediating effects testing.

## 4. Results

### 4.1. Reliability and validity

This study conducted a confirmatory factor analysis using AMOS 24.0. As shown in Table 3, our results suggest that the measurement model fit the data substantially (*χ*^2^/df = 1.262, CFI = 0.985, TLI = 0.983, RMSEA = 0.031, RMR = 0.050), confirming convergent validity. CR and Cronbach’s Alpha are all over 0.7 (Hair, 2009), all standardized factor loadings are over 0.5, AVE value (Fornell and Larcker, 1981). Table 2 shows that AVE obtained from every single construct met the acceptance level. Therefore, it is proved that this study has good convergent validity.

**Table 2:** Results of reliability and validity.

Construct	Items	Std. factor loadings	CR	AVE	Cronbach’s Alpha
Job crafting strengths	JCS1	0.838	0.887	0.662	0.886
	JCS2	0.844			
	JCS3	0.804			
	JCS4	0.767			
Job crafting interests	JCI1	0.849	0.938	0.751	0.930
	JCI2	0.819			
	JCI3	0.902			
	JCI4	0.900			
	JCI5	0.861			
Autonomous motivation	INTRINS	0.749	0.750	0.601	0.847
	INDENT	0.800			
Controlled motivation	INTRO	0.703	0.708	0.549	0.878
	EXT	0.777			
Innovative behavior	IB1	0.856	0.913	0.638	0.911
	IB2	0.769			
	IB3	0.895			
	IB4	0.787			
	IB5	0.728			
	IB6	0.742			

CFI, comparative fit index; TLI, Tucker-Lewis index; RMSEA, root mean square error of approximation; RMR, root mean square residual; CR, composite reliability; AVE, average variance extraction.

### 4.2. Discriminant validity and correlations

Typically, discriminant validity is assessed by comparing the squared correlations between two distinct weights in either construct, which should be less than the AVEs by the measures of a construct (Fornell and Larcker, 1981). The results of the discriminant validity test are shown in Table 3. All the square roots of AVEs exceeded the correlation between the constructs comprising each pair. Consequently, the constructs of this model have acceptable discriminant validity. In addition, it offered preliminary confirmation of hypotheses.

**Table 3:** Discriminant validity and correlations.

Variable	M	SD	1	2	3	4	5	6	7	8	9	10
1.Gender	1.34	0.474	–									
2.Age	2.18	0.913	−0.090	–								
3.Education	2.85	0.768	−0.014	−0.037	–							
4.Occupation	1.84	1.177	0.048	0.050	−0.008	–						
5.Income	3.99	1.434	−0.096	0.055	−0.094	−0.089	–					
6.JCS	3.87	0.890	−0.065	−0.024	0.006	−0.027	−0.097	0.886				
7.JCI	3.70	0.961	−0.036	−0.036	0.002	0.025	−0.063	0.538^**^	0.917			
8.AM	3.62	0.905	−0.019	0.003	−0.079	−0.146^*^	0.012	0.390^**^	0.455^**^	0.847		
9.CM	3.77	0.872	0.003	−0.078	−0.077	−0.095	−0.039	0.371^**^	0.394^**^	0.533^**^	0.878	
10.IB	3.72	0.929	−0.024	−0.045	−0.025	0.036	−0.078	0.514^**^	0.587^**^	0.486^**^	0.471^**^	0.911

**p < 0.01 and *p < 0.05; JCS, job crafting strengths; JCI, job crafting interests; AM, autonomous motivation; CM, controlled motivation; IB, innovative behavior; the diagonal value is the square roots of AVEs.

### 4.3. Common method bias

The purpose of Harman’s single-factor test was to examine the issue of common method bias. The analysis identified seven factors with eigenvalues greater than 1, with the first factor explaining less than 40% (Saether, 2019) of the variance (39.80% of 77.13%). Thus, the findings did not provide substantial evidence of common method bias.

### 4.4. Results of hypotheses testing

#### 4.4.1. Results of direct effects

**Figure 2:**
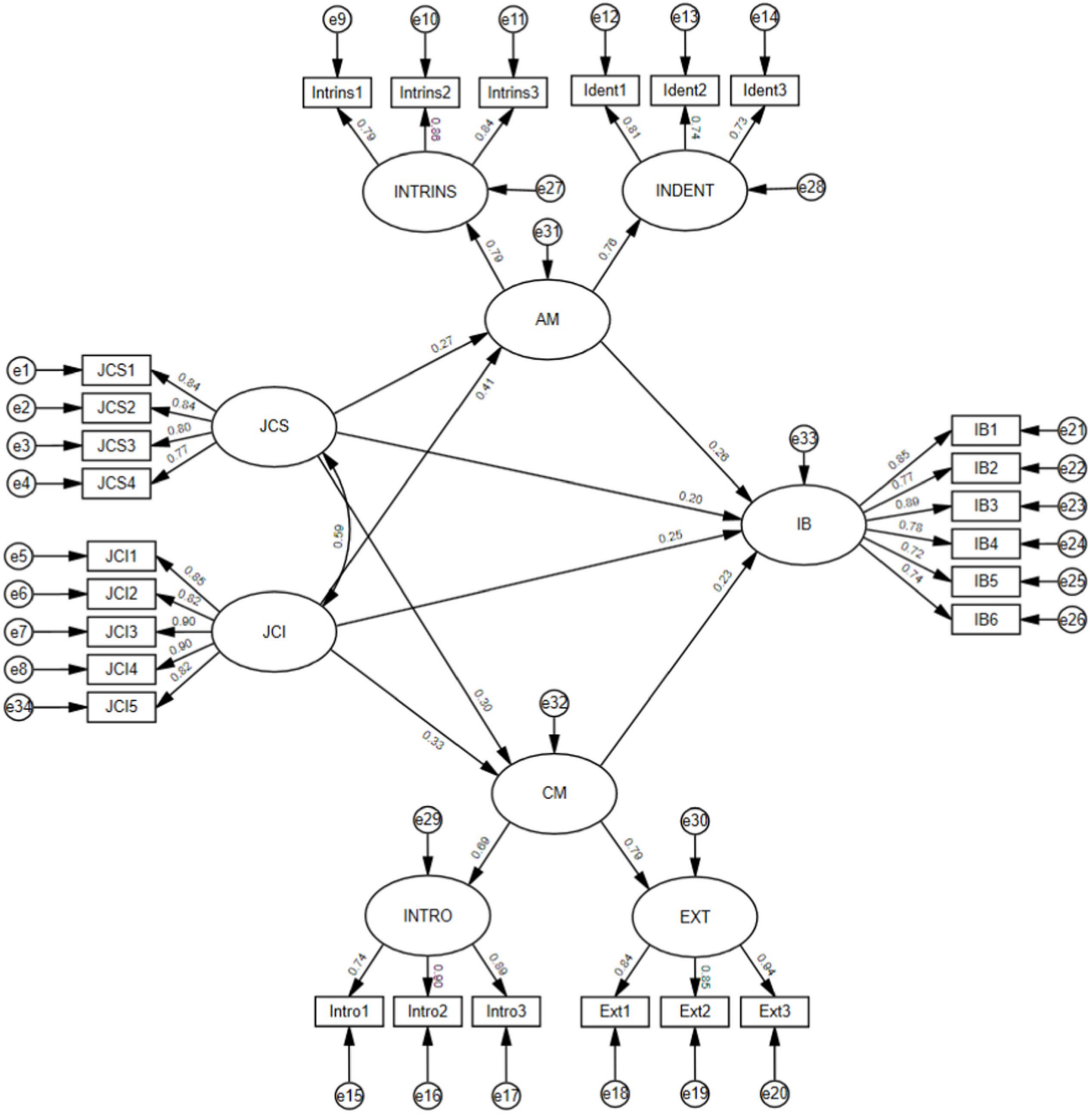
Structural model.

The SEM analysis (Table 4 and Figure 2) shows that job crafting strengths has a significant positive effect on autonomous motivation (*β* = 0.272, *p* < 0.05); job crafting interests has a significant positive effect on autonomous motivation (*β* = 0.414, *p* < 0.05); job crafting strengths has a significant positive effect on controlled motivation (*β* = 0.302, *p* < 0.05); job crafting interests has a significant positive effect on controlled motivation (*β* = 0.330, *p* < 0.05); autonomous motivation has a significant positive effect on innovative behavior (*β* = 0.258, *p* < 0.05); controlled motivation has a significant positive effect on innovative behavior (*β* = 0.232, *p* < 0.05). Thus, Hypothesis 1–6 were supported. In addition, although this study does not make the hypotheses of job crafting strengths on IB and job crafting interests on innovative behavior, the analysis results show that job crafting strengths also has a significant positive effect on innovative behavior, and job crafting interests has a significant positive impact on innovative behavior. Thus, hypotheses 1–6 are supported.

**Table 4:** Direct effects.

Path			Std. Estimate	Estimate	S.E.	*T*	*p*	Results
AM	<---	JCS	0.272	0.241	0.079	3.037	0.002	Supported
AM	<---	JCI	0.414	0.316	0.069	4.547	***	Supported
CM	<---	JCS	0.302	0.195	0.061	3.184	0.001	Supported
CM	<---	JCI	0.330	0.183	0.052	3.510	***	Supported
IB	<---	AM	0.258	0.305	0.099	3.071	0.002	Supported
IB	<---	CM	0.232	0.377	0.125	3.017	0.003	Supported
IB	<---	JCS	0.201	0.210	0.075	2.784	0.005	Supported
IB	<---	JCI	0.250	0.226	0.068	3.315	***	Supported

***p < 0.001; JCS, job crafting strengths; JCI, job crafting interests; AM, autonomous motivation; CM, controlled motivation; IB, innovative behavior. Model fit: *χ*^2^/df = 1.409, RMR = 0.094, TLI = 0.973, CFI = 0.976, RMSEA = 0.038.

#### 4.4.2. Results of mediating effects

Table 5 shows the results of indirect effects testing using AMOS 24.0. This study used bootstrapping 5,000 times resampling to observe whether the 95% confidence interval contains 0 to determine whether the mediating effects can exist. The bootstrap test results (Table 5) confirmed that there is a significant mediating effect of autonomous motivation on the relationship between job crafting strengths and innovative behavior (indirect effect = 0.070, Lower 0.008 − Upper 0.206), job crafting interests and innovative behavior (indirect effect = 0.107, Lower 0.026 − Upper 0.236); controlled motivation as a mediator has a significant impact on the relationship between job crafting strengths and innovative behavior (indirect effect = 0.070, Lower 0.007 − Upper 0.203), job crafting interests and innovative behavior (indirect effect = 0.077, Lower 0.011 − Upper 0.193). Thus, hypotheses 7–10 are supported.

**Table 5:** Indirect effects.

Path	Indirect effects	Bias-corrected 95% CI	
		Lower	Upper
JCS-AM-IB	0.070	0.008	0.206
JCI-AM-IB	0.107	0.026	0.236
JCS-CM-IB	0.070	0.007	0.203
JCI-CM-IB	0.077	0.011	0.193

JCS, job crafting strengths; JCI, job crafting interests; AM, autonomous motivation; CM, controlled motivation; IB, innovative behavior.

## 5. Discussion

Researchers and managers are interested in how job crafting influences employee innovative behavior (Grant and Parker, 2009; Zhang and Parker, 2019). According to previous research on job crafting, numerous new and distinct terms have emerged to describe the specific job crafting behavior (Zhang and Parker, 2019; Tims et al., 2022). This study encourages knowledge workers to use their strengths and interests in job crafting to generate innovative behavior through controlled and autonomous motivation. Consequently, we address these research voids by constructing a model of job strengths and job interests for knowledge workers’ innovative behavior in the gig economy using OIT as a foundation.

### 5.1. Theoretical implications

Our findings contribute in several ways to the existing literature on organismic integration theory and job design theory.

First, we highlighted how strengths and interests are crucial approaches for knowledge workers’ job crafting in the gig economy and how they promote work motivation, which is less apparent in existing empirical studies. In contrast to previous research (Zhang et al., 2021), our study reveals that interests are more important than strengths when it comes to job crafting. This unexpected result may be due to the fact that knowledge workers in the gig economy tend to tailor their occupations to their personal interests. Knowledge workers’ pursuit of interests, such as learning, teaching, and utilizing technology, can be a significant source of engagement and meaningfulness (Carbonneau et al., 2008; Csikszentmihalyi and Csikszentmihalyi, 2014; Kuijpers et al., 2020). This dearth of awareness of their strengths may be a contributing factor. For instance, employees may have difficulty recognizing their strengths if they believe that anyone is capable of possessing them (Buckingham and Clifton, 2001; Bakker and Van Woerkom, 2018).

On the other hand, the majority of employees will be aware of their passions, and incorporating them into their work may encourage innovative behavior. For instance, a relationship-building expert, such as a business consultant, could design his/her role of selling consulting services so that he/she speaks more candidly with each client as opposed to presenting to large groups (Kooij et al., 2017). Thus, knowledge workers develop a deeper understanding of their strengths and interests and a propensity to influence their environments to match their identities.

Consequently, one of this study’s central issues is the significance of pursuing autonomous and controlled motivations. In consistent with (Ratelle et al., 2007), it should be comprehensive consideration for knowledge workers to seek out jobs that they find inherently pleasurable (autonomous motivation) while paying attention to the extrinsic consequences (controlled motivation) of those jobs in the gig economy. Seeking only immediate pleasure (autonomous motivation) without regard for external regulations (controlled motivation) may significantly reduce future employment opportunities and outcomes. In contrast, focusing solely on extrinsic regulations and incentives can substantially inhibit innovative behavior. Moreover, this study confirmed the mediating role of motivation between job crafting and innovative behavior. Both autonomous and controlled motivation mediate the effect of job crafting on innovative behavior. Previous research findings have been empirically validated (Wrzesniewski and Dutton, 2001) that even in jobs with limited autonomy, knowledge workers can create new domains for mastery and modify aspects of job tasks to innovate in the gig economy. Overall, the mediating role of controlled or autonomous motivation between job crafting and innovative behavior can be essential in understanding how job crafting promotes innovative behavior in the gig economy. Meanwhile, the development of the gig economy informs our study of knowledge work trends in China.

### 5.2. Practical implications

Since job crafting is advantageous for both individuals and organizations, our findings also have significant implications for practice.

First, according to OIT, providing the necessary job resources to knowledge workers will promote the internalization of external motivation. Knowledge workers are highly skilled employees who have typically invested a considerable amount of time in formal education and professional development (Drucker, 1992). With years of specialization, it is likely that knowledge workers have an intrinsic interest in their profession (Markova and Ford, 2011). These employees’ theoretical and practical expertise is a valuable asset to their respective organizations. Organizations will be able to intervene more effectively to promote innovative behavior if they are aware that knowledge workers may engage in job crafting (Lee and Lee, 2018).

Second, theories and empirical research indicate that job-creating strengths and interests can promote innovative employee behavior through controlled and autonomous motivation. Self-motivation should be the primary objective of job-crafting for knowledge employees. Since knowledge workers perceive their work as a source of their identity, the job itself can be an effective source of intrinsic motivation (Giancola, 2011). While OIT provides the framework for the development of conceptual models, empirical findings also inform and expand the theory. When modelling job-crafting strengths and interests as predictors of controlled and autonomous motivation, the latter was a more direct predictor of these outcomes than the former, indicating that controlled and autonomous motivation mediated the relationship between job crafting and innovative behavior. Given that a central tenet of OIT is that extrinsic motivation can be subdivided into various forms based on the locus of causation or degree of internalization (Howard et al., 2017), the current findings are novel and significant. It appears that job-crafting interests explain why knowledge workers perform their jobs, while job-crafting strengths explain how they perform their jobs. The results elucidate the role of motivational internalization between job crafting and innovative behavior, a topic that is poorly understood in the current OIT literature.

Thirdly, this research responds to Locke and Schattke (2019) suggestion for a more differentiated motivational framework. OIT provides an appropriate framework for elucidating the propensity of individuals to internalize subjective motivations for innovative behavior. OIT implies an innate, natural tendency toward differentiation and integration that does not require external prodding and pressing. Not all change is intrinsically motivated; the only developmentally significant change is one that represents differentiating and integrating activity based on strengths or interests (Deci et al., 1985). This effective theoretical combination of OIT and job crafting theory provides a comprehensive road map for knowledge workers to engage in innovative behavior.

Overall, due to the rapid development of artificial intelligence, the gig economy will offer a variety of work forms and content, presenting knowledge workers with numerous opportunities and challenges and facilitating job crafting and behavior innovation among knowledge workers.

### 5.3. Limitations and future research

Despite the aforementioned implications, our research inevitably has several potential limitations, some of which may inspire future investigations. Although this study passed the test for common method bias (Podsakoff et al., 2003), there will be an issue with common method bias: all the variables were measured with a single questionnaire on a 5-point Likert scale. According to Deci et al. (2017), in order to accurately test causal relationships, future research should employ more longitudinal designs and more objective measures. Second, this study utilized only controlled and autonomous motivation as the relationship’s mediators, ignoring other variables that can moderate the management phenomenon. Therefore, future research can analyse this topic from a variety of theoretical perspectives in order to gain a deeper understanding of this management issue. This study focuses solely on the individual level of job crafting. Third, due to the convenience of online surveys for sampling, the sample is not gender-balanced (Male = 187, Female = 96). Future research should use random sampling for gender balance if possible.

Future research should investigate job crafting at both the individual and team levels in order to examine how individual and team levels impact motivation and can lead to exciting outcomes. In addition, future research can focus on combining knowledge workers and AI technology to understand how knowledge workers use AI technology to learn and develop new skills to maintain market competitiveness continuously and how organizations should benefit from knowledge workers and their knowledge to prepare for the gig economy.

## 6. Conclusion

We extend the research on innovative behavior by analyzing how knowledge workers can use their strengths and interests to design their employment, resulting in controlled and autonomous motivation. There is reason to believe that the processes investigated in this study are even more important for knowledge workers in the freelance economy. The gig economy has profoundly altered organizations’ and individuals’ preexisting perceptions and behaviors. Consequently, societal changes, specifically, shifts in how people define “more important work” and “less important work” (Kramer and Kramer, 2020) could influence the propensity for innovation among knowledge workers. On the basis of OIT, it was found that motivation can increase innovative behavior. Nevertheless, job-crafting interests have a greater impact on autonomous motivation, controlled motivation, and innovative behavior than do strengths. Our findings supported the universality and applicability of the OIT to Chinese knowledge workers, especially those in the contract economy. Specifically, controlled and autonomous motivation were identified as the variables that mediated the relationship between job fabrication and innovative behavior. On the other hand, the data highlighted the significance of the mechanism’s ability to identify and disseminate effective management practices.

## Data availability statement

The raw data supporting the conclusions of this article will be made available by the authors, without undue reservation.

## Ethics statement

The requirement of ethical approval was waived by Gachon University for the studies involving humans because the study did not collect or record ‘sensitive information’ from the participants. The studies were conducted in accordance with the local legislation and institutional requirements. Written informed consent was obtained from the individual(s) for the publication of any potentially identifiable images or data included in this article.

## Author contributions

All authors listed have made a substantial, direct, and intellectual contribution to the work and approved it for publication.

## Conflict of interest

The authors declare that the research was conducted in the absence of any commercial or financial relationships that could be construed as a potential conflict of interest.

## Publisher’s note

All claims expressed in this article are solely those of the authors and do not necessarily represent those of their affiliated organizations, or those of the publisher, the editors and the reviewers. Any product that may be evaluated in this article, or claim that may be made by its manufacturer, is not guaranteed or endorsed by the publisher.
